# Identification of endophytic fungi from leaves of Pandanaceae based on their morphotypes and DNA sequence data from southern Thailand

**DOI:** 10.3897/mycokeys.33.23670

**Published:** 2018-03-28

**Authors:** Saowaluck Tibpromma, Kevin D. Hyde, Jayarama D. Bhat, Peter E. Mortimer, Jianchu Xu, Itthayakorn Promputtha, Mingkwan Doilom, Jun-Bo Yang, Alvin M. C. Tang, Samantha C. Karunarathna

**Affiliations:** 1 Key Laboratory for Plant Diversity and Biogeography of East Asia, Kunming Institute of Botany, Chinese Academy of Science, Kunming 650201, Yunnan, People’s Republic of China; 2 Centre of Excellence in Fungal Research, Mae Fah Luang University, Chiang Rai, 57100, Thailand; 3 Formerly, Department of Botany, Goa University, Taleigão, Goa, India; 4 No. 128/1-J, Azad Housing Society, Curca, Goa Velha, India; 5 Department of Biology, Faculty of Science, Chiang Mai University, Chiang Mai, 50200, Thailand; 6 Environmental Science Research Centre, Faculty of Science, Chiang Mai University, 50200, Thailand; 7 Germplasm Bank of Wild Species in Southwest China, Kunming Institute of Botany, Chinese Academy of Science, Kunming 650201, Yunnan, China; 8 Division of Applied Science, College of International Education, The Hong Kong Baptist University, Hong Kong SAR, China

**Keywords:** Ascomycetes, Basidiomycota, biodiversity, phylogenetic analysis

## Abstract

The authors established the taxonomic status of endophytic fungi associated with leaves of Pandanaceae collected from southern Thailand. Morphotypes were initially identified based on their characteristics in culture and species level identification was done based on both morphological characteristics and phylogenetic analyses of DNA sequence data. Twenty-two isolates from healthy leaves were categorised into eight morphotypes. Appropriate universal primers were used to amplify specific gene regions and phylogenetic analyses were performed to identify these endophytes and established relationships with extant fungi. The authors identified both ascomycete and basidiomycete species, including one new genus, seven new species and nine known species. Morphological descriptions, colour plates and phylogenies are given for each taxon.

## Introduction

Endophytic fungi are beneficial to their host plants and have the ability to produce bioactive compounds that have applied uses (Fisher et al. 1994; Strobel et al. 2004; Gunatilaka 2006; Arnold et al. 2007; Saikkonen et al. 2010; Aly et al. 2010; Lin et al. 2010; Rajulu et al. 2011; Chowdhary et al. 2015). Research on endophytic fungi began approximately 30 years ago and has intensified over the past 20 years (Thomson et al. 1997; Arnold et al. 2000; Stone et al. 2000; Hyde and Soytong 2008; Lumyong et al. 2009). This rising interest in endophytic fungi dates back to Bills’ 1996 novel concept that mycelia sterilia isolates could be assigned to groups based on their degree of similarity in colony surface texture (Rodrigues 1994; Fisher et al. 1995; Lodge et al. 1996; Brown et al. 1998; Taylor et al. 1999; Umali et al. 1999; Fróhlich et al. 2000). Lacap et al. (2003) used molecular data to demonstrate the reliability of Bill’s 1996 concept based on the cultural approach. Guo et al. (2000, 2003) found that morphological characteristics were insufficient to identify most endophyte isolates, especially when they do not sporulate and so DNA sequence data were used for identification of these taxa. Although this has been followed by numerous authors using ITS sequence data analysis, the use of ITS alone is not accurate (Promputtha et al. 2005). Subsequent studies have shown that multi-gene analyses are needed to identify endophytes (Ko et al. 2011).

Endophytic fungal strains have been isolated from many different plants including trees, vegetables, fruits, cereal grains and other crops (Rosenblueth and Martinez-Romero 2006). Dickinson (1976) published the first study of endophyte - leaf associations. However, there has been less research on the endophytic fungi associated with the leaves of tropical plants (Promputtha et al. 2007). The high species diversity of endophytic fungi makes their study a pressing research area. Globally, endophytic fungi were estimated to comprise 7 % of the 1.5 million species of fungi (Hawksworth 2001; Chowdhary et al. 2015). The actual numbers may be far higher. Recently, Hawksworth and Lucking (2017) estimated that there are 2.2 to 3.8 million fungal taxa. Endophytes are expected to be numerous because their host-specificity will drive diversification and they can occupy several niches, including that of pathogens and saprobes (Zhou and Hyde 2001). Several studies have investigated the relationships between endophytes and saprotrophs and also between endophytes and pathogens (Petrini 1991; Yanna and Hyde 2002; Ghimire and Hyde 2004; Photita et al. 2004; Hyde et al. 2006).

The authors have been investigating saprobic and endophytic fungi associated with Pandanaceae (Tibpromma et al. 2016a, b, c, 2017a, b) and, in this study, taxonomic details are presented regarding the endophytic fungi that were isolated. Pandanaceae are monocotyledonous plants. Their associated endophytic fungi were first studied by McKenzie et al. (2002), with further research conducted by Thongkantha et al. (2008), Bungihan et al. (2011), Ariffin (2013), Bungihan et al. (2013) and Eskandarighadikolaii et al. (2015).

The objectives of the present study were to establish the endophytic fungal community on selected Pandanaceae collected in southern Thailand. The authors isolated 22 endophytic isolates and sorted them in morphotypes and identified the taxa based on DNA sequence analyses. Both ascomycete and basidiomycete genera were identified, including one new genus, seven new species and nine known species. The recommendations of Jeewon and Hyde (2016) were followed when introducing the new species based on molecular data.

## Materials and methods

### Sample collection and fungal isolation

**Figure 1.**
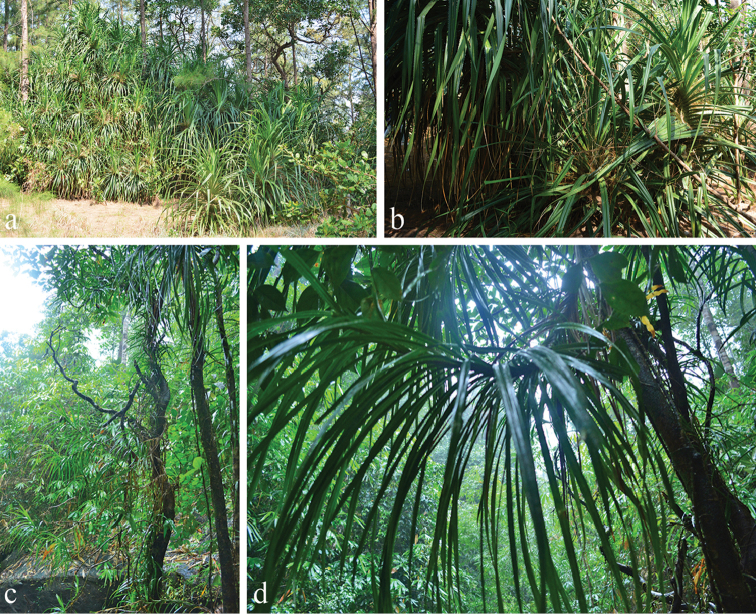
Habitats of the host plants: **a, b** *Pandanus* spp. **c, d** *Freycinetia* spp.

Healthy mature leaves of *Pandanus* and *Freycinetia* species (Pandanaceae, Figure 1) were collected from Chumphon (10°57'38.2"N 99°29'21.8"E) and Ranong (9°55'15.9"N, 98°38'30.7"E) provinces of southern Thailand during the rainy season (December) of 2016. Leaves with physical damage or showing signs of pathogenic infection were excluded from the study. In total, more than 100 healthy leaves were placed in Ziploc plastic bags, preserved with ice and transported to the laboratory. Leaves were randomly cut into 0.5 cm size pieces (10 pieces/leaf) using a hole puncher under aseptic conditions. These sections were soaked in 95 % ethanol for 1 minute, then in 3 % sodium hypochlorite solution for 3 minutes and finally in 95 % ethanol for 30 seconds. All samples were rinsed with sterile distilled water and dried on sterile tissue paper. Leaf sections were placed in Malt Extract Agar (MEA), Potato Dextrose Agar (PDA) and Water Agar (WA). They were incubated at room temperature (25-30 °C) for 1-3 days. If hyphal tips of any fungal colony appeared during incubation, the colony was transferred to new PDA plates and incubated to obtain pure cultures.

### Cultures and identification

The above methods resulted in 22 isolates which were separated into morphotypes based on visual assessment of the similarity of the cultures (Bills 1996; Umali et al. 1999; Fróhlich et al. 2000; Lacap et al. 2003). All of these cultures were grown on Potato Dextrose Agar (PDA). Growth rate measurements are shown in Table 1 with colony colour defined with the Methuen Handbook of Colour (Kornerup and Wanscher 1967). New taxa were examined in pure culture, allowing photographs, records of morphological characteristics and descriptions to be recorded. Herbarium specimens were prepared from cultures that were dried in silica gel. The holotypes were deposited in the Mae Fah Luang University Herbarium (Herb. MFLU), Chiang Rai, Thailand and in the Kunming Institute of Botany Academia Sinica (HKAS), Kunming, China. The ex-types cultures were deposited in the Mae Fah Luang University Culture Collection (MFLUCC) with duplicates deposited in the BIOTEC Culture Collection Laboratory (BCC) and the Kunming Institute of Botany Culture (KMUCC). New taxa were registered in Facesoffungi (FoF) (Jayasiri et al. 2015) and MycoBank (Crous et al. 2004).

**Table 1.** Details of genes/loci with PCR primers and protocols.

Gene/Loci	PCR primers (Forward/Reverse)	References
LSU	LROR/LR5	Vilgalys and Hester 1990
ITS	ITS5/ITS4	White et al. 1990
SSU	NS1/NS4	White et al. 1990
TEF1	983F/2218	Rehner 2001
	728F/986R	Carbone and Kohn 1999
RPB2	fRPB2-5f/fRPB2–7cR	Liu et al. 1999
β-tubulin	BT2a/BT2b	Glass and Donaldson 1995
	T1/T2	O’Donnell and Cigelnik 1997
Actin	512F/783R	Carbone and Kohn 1999
CHS-1	79F/354R	Carbone and Kohn 1999
GADPH	Gpd1/Gpd2	Myllys et al. 2002
	GDF/GDR	Templeton et al. 1992

### DNA extraction, PCR amplification, and sequencing

Genomic DNA was extracted from pure fungal cultures using Biospin Fungal Genomic DNA extraction Kit–BSC14S1 (BioFlux, P.R. China). Polymerase chain reaction (PCR) was used to amplify partial gene regions of Internal Transcribed Spacers (ITS), 28S ribosomal RNA (LSU), 18S ribosomal RNA (SSU), RNA polymerase II second largest subunit (RPB2), β-tubulin (Tub2), Actin (ACT), Glyceraldehyde-3- Phosphate Dehydrogenase (GADPH), Chitin synthase 1 (CHS-1) and Translation Elongation Factor 1-alpha (TEF1) using primers as shown in Table 1. The total volume of PCR mixtures for amplifications were 25 μl containing 8.5 μl ddH_2_O, 12.5 μl 2× Easy Taq PCR Super Mix (mixture of Easy Taq TM DNA Polymerase, dNTPs and optimised buffer (Beijing Trans Gen Biotech Co., Chaoyang District, Beijing, PR China), 2 μl of DNA template, 1 μl of each forward and reverse primers (10 pM). The quality of PCR products was checked on 1 % agarose gel electrophoresis stained with 4S green nucleic acid (Life Science Products & Services, Cat. No: A616694). Purification and sequencing of PCR products were carried out by Sangon Biotech Co., Shanghai, China.

### Phylogenetic analysis

The sequence data generated during this study were the subject of BLAST searches in the nucleotide database of GenBank (www http://blast.ncbi.nlm.nih.gov/) to determine their most probable closely related taxa. Sequence data were retrieved from GenBank based on recent publications. Raw forward and reverse sequences were assembled using Geneious Pro.v4.8.5. Sequence alignments were carried out with MAFFT v.6.864b (Katoh and Standley 2016) and alignments were manually improved where necessary. The sequence datasets were combined using BioEdit v.7.2.5 (Hall 2004). Maximum Likelihood (ML) and Bayesian Inference (BI) analyses were performed for the sequence dataset. The phylogenetic trees were configured in FigureTree v. 1.4 (Rambaut and Drummond 2008) and edited using Microsoft Office PowerPoint 2007 and Adobe Illustrator CS3 (Adobe Systems Inc., USA).

## Results and discussion

### Identification of morphotypes

Twenty-two fungal isolates from *Pandanus* and *Freycinetia* species were recovered and these mycelia sterilia were separated into eight morphotypes based on the similarity of their culture characteristics, as summarised in Table 2 (Bills 1996; Umali et al. 1999; Fróhlich et al. 2000; Lacap et al. 2003).

**Table 2.** Culture characteristics of the 22 strains (8 morphotypes) of mycelia sterilia on PDA.

Morpho-types	Isolate code	Host	Size (cm) of colony		Shape	Colour		Mycelium	Edge
			3 days	7 days		Above	Reverse		
1	PE05	*Pandanus* sp.	4.6	>A	Circular	4A1	4A2	Aerial	Undulate
	PE09	*Pandanus* sp.	4.6	>A	Circular	6D3	6B3	Aerial	Entire
	PE15	*Pandanus* sp.	>A	>A	Circular	5B2	5B3	Flat	Entire
2	PE10	*Pandanus* sp.	1.1	3.2	Irregular	4C1	4A3	Aerial	Undulate
	PE60	*Pandanus* sp.	1.6	3.8	Irregular	4B1	4A3	Aerial	Undulate
	FE46	*Freycinetia* sp.	2.1	5.6	Irregular	5B2	5A2	Aerial	Undulate
	FE42	*Freycinetia* sp.	1.5	5	Irregular	4A1	4A3	Aerial	Undulate
	FE43	*Freycinetia* sp.	1.4	4.2	Irregular	5D4	5C4	Flat	Undulate
	PE75	*Pandanus* sp.	1.6	5	Circular	6A1/6D3	6A1/6F5	Aerial	Undulate
	PE84	*Pandanus* sp.	1.5	3.8	Circular	5F4	5F7	Aerial	Curled
	FE98	*Freycinetia* sp.	1.3	3.1	Irregular	5B2	5D5	Flat	Filamentous
3	PE25	*Pandanus* sp.	>A	>A	Circular	5E1	5F2	Aerial	Entire
4	PE26	*Pandanus* sp.	3.1	7.2	Irregular	5B3	5B5	Aerial	Undulate
	PE52	*Pandanus* sp.	1.2	2.9	Circular	5A2	5A3	Aerial	Undulate
5	PE35	*Pandanus* sp.	1.1	2.7	Filamentous	8E2	8F2	Aerial	Filamentous
6	PE92	*Pandanus* sp.	5.1	>A	Irregular	4B1	4A6	Aerial	Curled
	PE37	*Pandanus* sp.	2.3	7.9	Circular	4A1	4B3	Aerial	Curled
	FE88	*Freycinetia* sp.	2.9	6.2	Circular	5D3	5B2	Flat	Undulate
	PE77	*Pandanus* sp.	4.2	7.1	Irregular	6B1/6E1	6B2	Aerial	Undulate
	FE41	*Freycinetia* sp.	>A	>A	Irregular	4D2	4F6	Flat	Filamentous
7	PE58	*Pandanus* sp.	<B	1.7	Circular	4F2	4F8	Aerial	Entire
8	FE101	*Freycinetia* sp.	<B	2	Circular	4B2	4A3	Aerial	Entire

Notes: >A Completely covering plate, <B Less than 1 cm

### Phylogenetic analysis

Based on phylogenetic analysis, 22 fungal isolates were identified for 16 species. These include one new genus, seven new species and nine known species. All sequences obtained from this study are summarised in Table 3.

**Table 3.** Species of endophytes obtained in this study.

No.	Original code	Species name	Culture collection no.
1	PE26	*Alternaria burnsii*	MFLUCC 17-0582
2	PE58	*Cladosporium endophyticum*	MFLUCC 17-0599
3	PE09	*Colletotrichum pandanicola*	MFLUCC 17-0571
4	FE88	*Colletotrichum fructicola*	MFLUCC 17-0555
	PE84		MFLUCC 17-0613
5	PE77	*Diaporthe pandanicola*	MFLUCC 17-0607
6	PE37	*Diaporthe siamensis*	MFLUCC 17-0591
7	FE41	*Endomelanconiopsis freycinetiae*	MFLUCC 17-0547
8	FE42	*Endopandanicola thailandica*	MFLUCC 17-0548
	FE43		MFLUCC 17-0549
	FE46		MFLUCC 17-0551
	PE10		MFLUCC 17-0572
	PE60		MFLUCC 17-0600
9	PE25	*Lasiodiplodia theobromae*	MFLUCC 17-0581
10	PE52	*Massarina pandanicola*	MFLUCC 17-0596
11	FE98	*Meyerozyma caribbica*	MFLUCC 17-0556
	PE75		MFLUCC 17-0606
12	FE101	*Mycoleptodiscus endophytica*	MFLUCC 17-0545
13	PE05	*Pestalotiopsis jiangxiensis*	MFLUCC 17-0567
14	PE92	*Pestalotiopsis microspora*	MFLUCC 17-0619
15	PE15	*Phanerochaete chrysosporium*	MFLUCC 17-0575
16	PE35	*Phyllosticta capitalensis*	MFLUCC 17-0589

### Basidiomycota R.T. Moore

#### Agaricomycetes Doweld

##### Polyporales Gäum., 1926

###### Polyporaceae

Taxon classificationFungiPolyporalesPolyporaceae

Fr. ex Corda

####### Remarks.

The family *Polyporaceae* was introduced by Fr. ex Corda (1839) and includes 92 genera and 636 species (Kirk et al. 2008). According to Cannon and Kirk (2007), the species in this family are characterised by poroid, irregular or lamellate hymenophores and are saprobes. Recent phylogenetic analyses of *Polyporaceae* are by Binder et al. (2013) and Hyde et al. (2017). In this study, a new endophytic genus, *Endopandanicola* with *En. thailandica* as the type species was discovered. In addition to the new genus, *Phanerochaete chrysosporium* was also identified.

###### Endopandanicola

Taxon classificationFungiPolyporalesPolyporaceae

Tibpromma & K.D. Hyde gen. nov.

MB823835

####### Etymology.

Named after its habitat as an endophyte of *Pandanus*.

####### Type species.

*Endopandanicola thailandica* Tibpromma & K.D. Hyde

####### Culture characteristics.

Colonies on PDA (PE60), superficial, initially white, later becoming yellow-white, smooth at the surface, irregular, with undulate margin, flossy to velvety; reverse white to yellow-white. Generative hyphae simple-septate, branched, sub-hyaline, thin-walled.

####### Notes.

*Endopandanicola* formed a single, well-supported clade (100 % in ML, 100 % in MP), which is distinct as compared to other genera in *Polyporaceae* (Figure 3). This genus comprises resupinate or crust polypores that live inside leaves or wood as endophytes and do not form fruiting bodies (sexual morph), but form flat mycelia. More collections of *Pandanus* are needed in the future to locate the sexual morph of *Endopandanicola*.

###### Endopandanicola thailandica

Taxon classificationFungiPolyporalesPolyporaceae

Tibpromma & K.D. Hyde sp. nov.

MB823836

Figure 4

####### Etymology.

named after Thailand, the country where the fungus was first discovered.

####### Holotype.

MFLU 18-0021

####### Culture characteristics.

**Figure 2.**
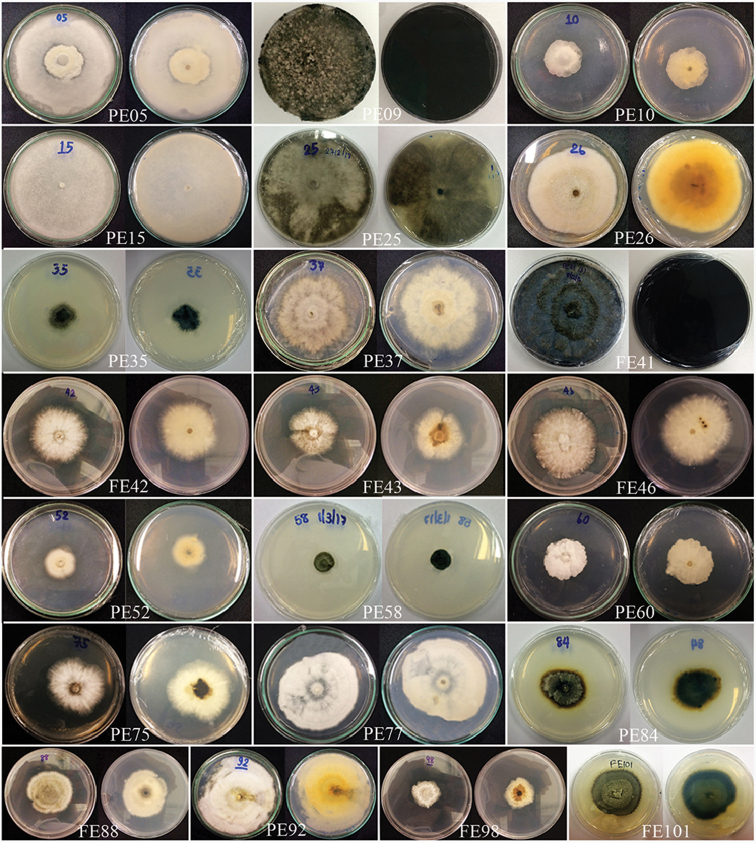
All cultures from this study are grown on PDA at room temperature after 7 days (original codes are written at the bottom of each picture).

Colonies on PDA (Figure 2 PE10, FE42, FE43, FE46 and PE60), superficial, initially white, later becoming yellow-white, irregular, with undulate margin, smooth with flossy to velvety; reverse white to yellow-white. Generative hyphae simple-septate, branched, with clamp connections, sub-hyaline, thin-walled, 1.5–3.5 µm wide.

####### Material examined.

THAILAND, Chumphon, Pathio District, on healthy leaves of *Pandanus* sp. (Pandanaceae), 1 December 2016, S. Tibpromma PE60 (MFLU 18-0021, **holotype**); HKAS100856, paratype, ex-type living cultures, MFLUCC 17-0600 = KUMCC 17-0295; Chumphon, Pathio District, 1 December 2016, S. Tibpromma PE10, living culture, MFLUCC 17-0572; Ranong, Muang, Muang District, 3 December 2016, S. Tibpromma FE42, living culture, MFLUCC 17-0548; FE43, living culture, MFLUCC 17-0549 = KUMCC 17-0264; FE46, living culture, MFLUCC 17-0551 = KUMCC 17-0265.

####### GenBank numbers.

ITS; MFLUCC 17-0545=MG646961, MFLUCC 17-0548=MG646964, MFLUCC 17-0549=MG646963, MFLUCC 17-0551=MG646962, MFLUCC 17-0572=MG646959, MFLUCC 17-0600=MG646960.

####### Notes.

**Figure 3.**
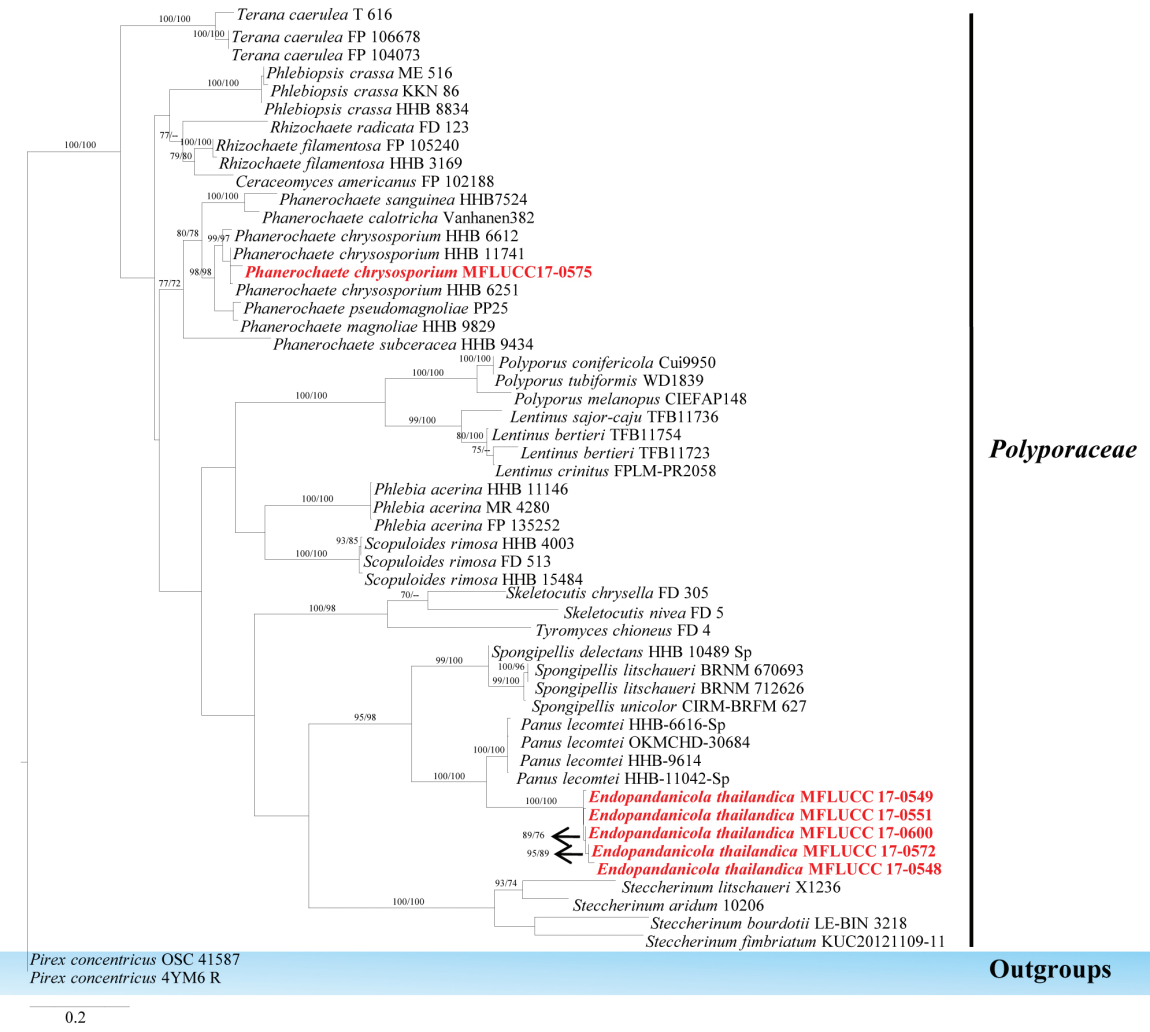
Phylogram generated from maximum likelihood analysis based on ITS sequence data. Maximum parsimony (left) and maximum likelihood (right) bootstrap support values are given above/below the nodes. The newly generated sequences are in red text. The tree is rooted with *Pirex concentricus*.

*Endopandanicola* is introduced and typified by *En. thailandica* which is represented by six isolates and is described as a novel species based on its asexual morph. The phylogenetic analysis of ITS sequence data showed that this species clustered together with *Panus*, but there is a high level of statistical support for its separation (100% in ML, 100% in MP) (Figure 3).

**Figure 4.**
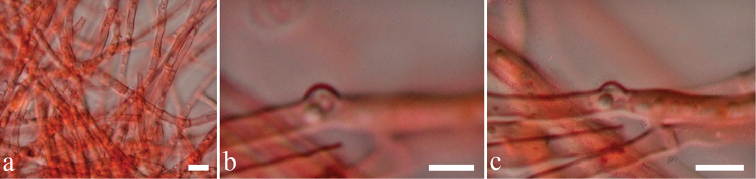
*Endopandanicola thailandica* (MFLU 18-0021, holotype). **a** Mycelia masses **b, c** Clamp connections. Scale bars: 10 μm (**a**), 5 μm (**b, c**).

###### Phanerochaete chrysosporium

Taxon classificationFungiPolyporalesPhanerochaetaceae

Burds., in Burdsall & Eslyn, Mycotaxon 1(2): 124 (1974)

####### Culture characteristics.

Colonies on PDA (Figure 2, PE15), superficial, white, surface smooth with flat media surface, circular, with entire edge; reverse white to yellow-white.

####### GenBank numbers.

ITS=MG646957.

####### Notes.

Burdsall and Eslyn (1974) introduced *Phanerochaete chrysosporium* which was collected on dead wood of *Platanus wrightii* in the USA. Phylogenetic analysis of ITS sequence data shows this taxon groups with *Phanerochaete chrysosporium* (sequences obtained from GenBank) that had been collected from different hosts. The phylogenetic placement of this species is shown in Figure 3.

### Ascomycota Whittaker

#### Dothideomycetes O.E. Erikss. & Winka

##### Botryosphaeriales

Taxon classificationFungiBotryosphaeriales

C.L. Schoch, Crous & Shoemaker

###### Remarks.

The order *Botryosphaeriales* was introduced by Schoch et al. (2006) with Botryosphaeriaceae as the type family. *Botryosphaeriales* is a diverse order with a worldwide distribution, comprising species that vary from endophytes to pathogens (Slippers and Wingfield 2007; Phillips et al. 2013; Chethana et al. 2016; Daranagama et al. 2016; Dissanayake et al. 2016; Konta et al. 2016a, b; Linaldeddu et al. 2016a, b, c; Manawasinghe et al. 2016; Zhang et al. 2017). Currently, nine families are recognised, namely, Aplosporellaceae, Botryosphaeriaceae, Endomelanconiopsisaceae, Melanopsaceae, Phyllostictaceae, Planistromellaceae, Pseudofusicoccumaceae, Saccharataceae and Septorioideaceae (Schoch et al. 2006; Minnis et al. 2012; Wikee et al. 2013; Slippers et al. 2013; Wyka and Broders 2016; Dissanayake et al. 2016; Yang et al. 2017). In this study, *Endomelanconiopsis freycinetiae* is introduced as a new species and reports are provided on *Phyllosticta capitalensis* and *Lasiodiplodia theobromae*.

##### Endomelanconiopsis freycinetiae

Taxon classificationFungiBotryosphaerialesBotryosphaeriaceae

Tibpromma & K.D. Hyde sp. nov.

MB823837

Figure 6

###### Etymology.

name referring to the host genus on which the fungus was found (*Freycinetia*).

###### Holotype.

MFLU 18-0002

###### Culture characteristics.

Colonies on PDA (Figure 2, FE41), superficial, initially white-grey with flat mycelium on media with dark centre, later becoming dark olivaceous with circular rings and flossy at the margin; reverse dark olivaceous. Generative hyphae simple-septate, branched, sub-hyaline to brown, cylindrical, guttulate, thick-walled. Not sporulating in culture (Figure 6).

###### Material examined.

THAILAND, Ranong, Muang, on healthy leaves of *Freycinetia* sp. (Pandanaceae), 3 December 2016, S. Tibpromma FE41 (MFLU 18-0002, holotype); HKAS100853, paratype, ex-type living cultures, MFLUCC 17-0547 = KUMCC 17-0292.

###### GenBank numbers.

ITS=MG646955, LSU=MG646948, TEF1=MG646983, β-tubulin=MG646924.

###### Notes.

*Endomelanconiopsis freycinetiae* is closely related to the endophytic fungus *En. endophytica*. Therefore, the culture characteristics of these two taxa were compared and it was found that, in *En. endophytica*, at first the hyphae are colourless, immersed, later becoming olivaceous in the centre with irregular concentric rings; aerial mycelia are dark olivaceous or grey when dense; shiny black when the aerial mycelia are loose (Rojas et al. 2008) whereas aerial mycelia of *En. freycinetiae* has dark olivaceous, circular rings and flossy surface (Figure 2, FE41). Nucleotide base pairs of ITS and TEF1 were also compared and it was found that there are differences (ITS 3 bp, TEF1 8 bp).

##### Phyllosticta capitalensis

Taxon classificationFungiBotryosphaerialesPhyllostictaceae

Henn., Hedwigia 48: 13 (1908)

###### Culture characteristics.

Colonies on PDA (Figure 2, PE35), superficial, dark olivaceous with filamentous hyphae and raised edge; reverse dark olivaceous. Sporulating in culture after 2 months.

###### GenBank numbers.

ITS=MG646954, LSU=MG646953, TEF1=MG646982.

###### Notes.

**Figure 5.**
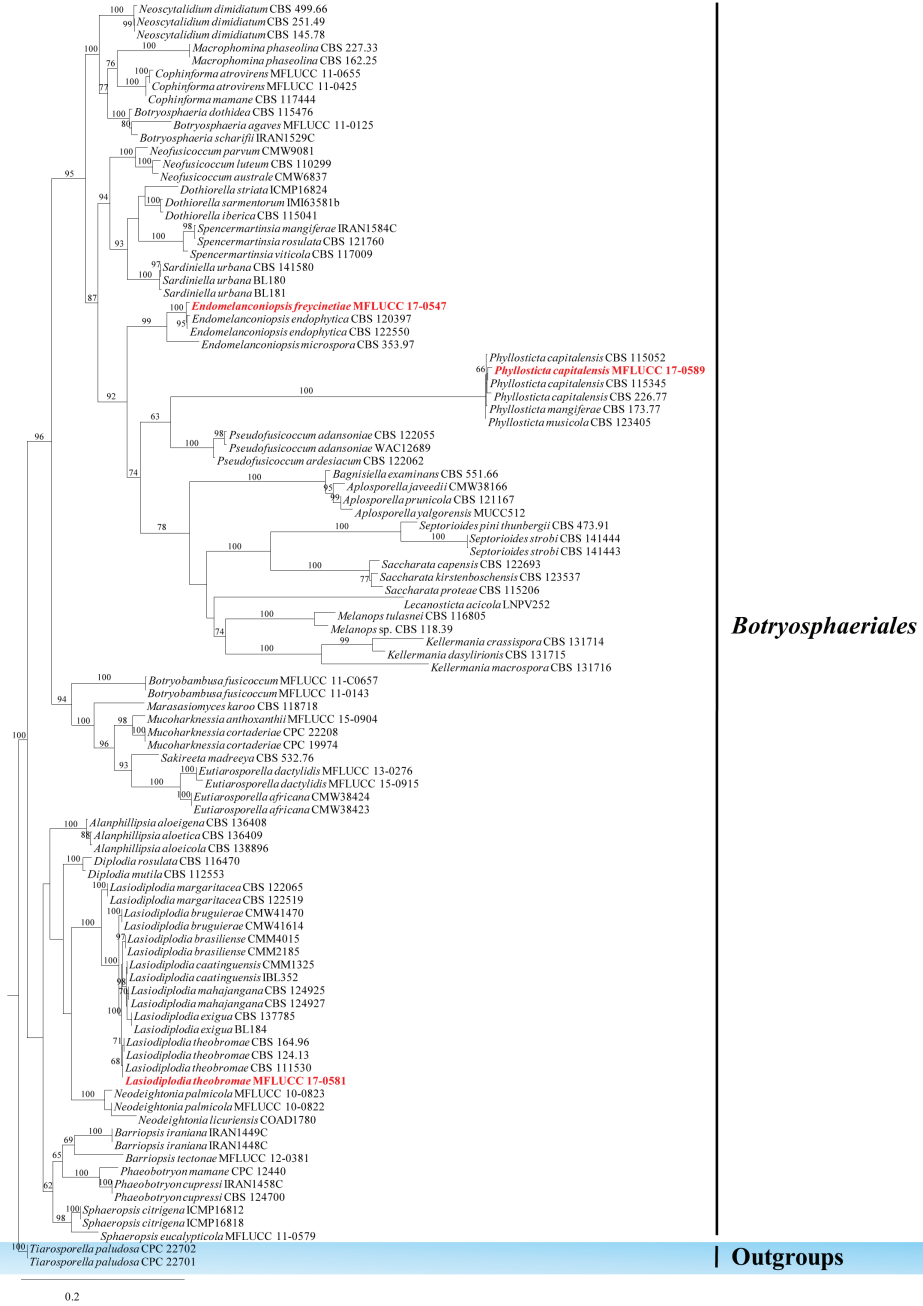
Phylogram generated from maximum likelihood analysis based on ITS, LSU and TEF1 sequenced data. Maximum likelihood bootstrap values are given above/below the nodes. The newly generated sequences are in red bold. The tree is rooted with *Tiarosporella paludosa*.

*Phyllosticta capitalensis* (Hennings 1908) is known as an endophytic taxon and a minor plant pathogen. It has a worldwide distribution and has been recorded on 70 plant families (Baayen et al. 2002; Okane et al. 2003; Motohashi et al. 2009; Wikee et al. 2013). The isolate recovered herein clusters with reasonable ML bootstrap support with other *P. capitalensis* isolates (Figure 5). Morphological examination also depicts similar morphs and hence it is identified as *P. capitalensis*.

**Figure 6.**
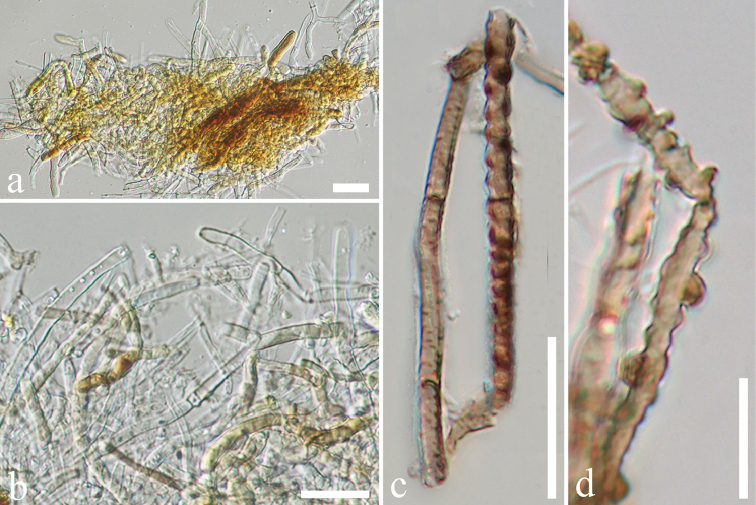
*Endomelanconiopsis freycinetiae* (MFLU 18-0002, holotype). **a–d** Mycelia masses. Scale bars: 20 μm (**a–c**), 10 μm (**d**).

##### Lasiodiplodia theobromae

Taxon classificationFungiBotryosphaerialesBotryosphaeriaceae

(Pat.) Griffon & Maubl., Bull. Soc. Mycol. Fr. 25: 57 (1909)

###### Culture characteristics.

Colonies on PDA (Figure 2, PE25), superficial, initially white with flat mycelium on media, later becoming dark, circular, flossy and velvety; reverse dark. Not sporulating in culture.

###### GenBank numbers.

ITS=MG646970, LSU=MG646945, SSU=MG646976, TEF1=MG646984.

###### Notes.

Morphological and phylogenetic data supported placement of this isolate as *Lasiodiplodia theobromae*. The phylogenetic analysis showed the isolate groups with *Lasiodiplodia theobromae*. Nucleotide base pairs of published sequences of *Lasiodiplodia theobromae* (strain EucN188, CBS 111530, PHLO9, CDFA145) were also compared with the sequence and found that the nucleotide base pairs of the ITS gene are 100% similar.

##### Capnodiales Woron., 1925

###### Cladosporiaceae Castell. & R.G. Archibald

####### Cladosporium

Taxon classificationFungiCapnodialesCladosporiaceae

Link, 1816

######## Remarks.

**Figure 7.**
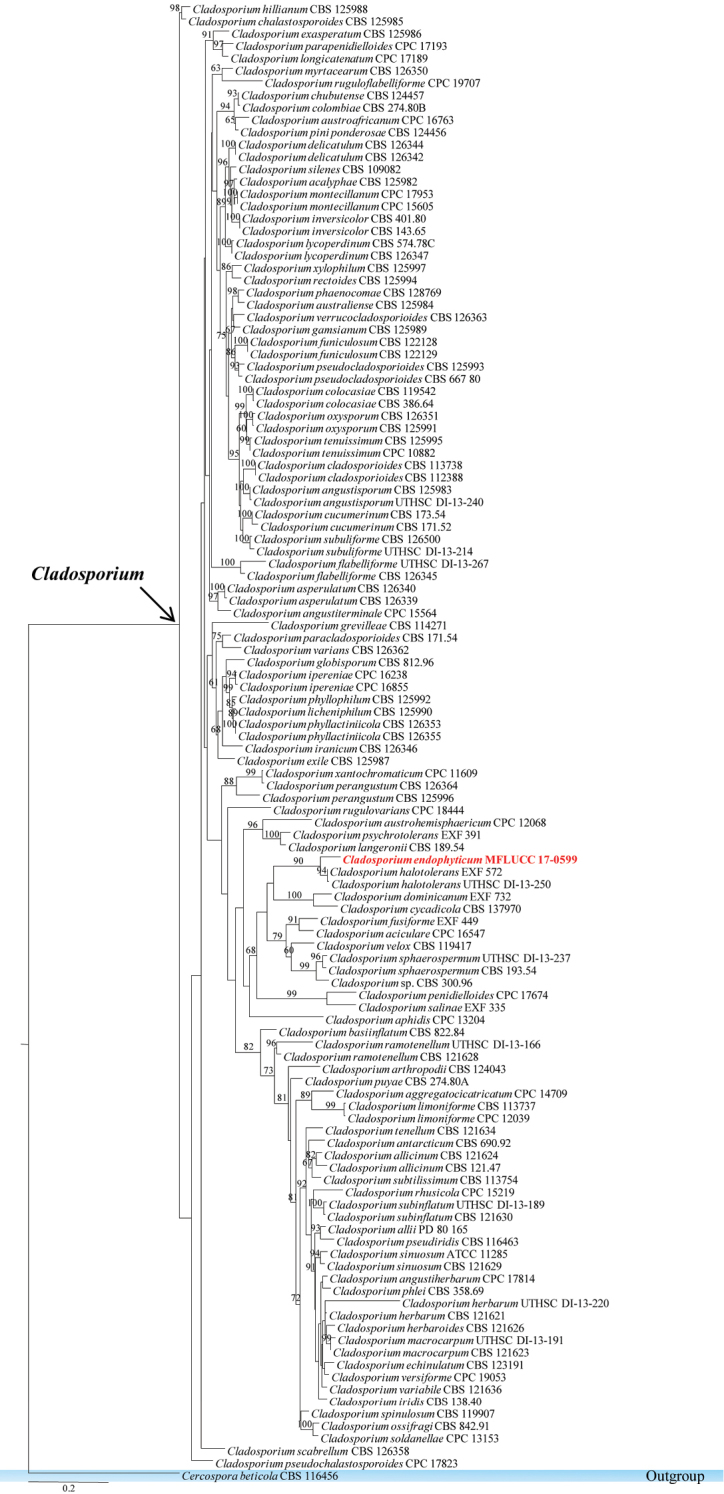
Phylogram generated from maximum likelihood analysis based on ITS, TEF1 and Actin sequenced data. Maximum likelihood bootstrap is given above/below the nodes. The newly generated sequences in red bold. The tree is rooted with *Cercospora beticola*.

The genus *Cladosporium* (*Cladosporiaceae*, *Capnodiales*) is a large genus of the Ascomycota (Wijayawardene et al. 2017). The genus comprises species that are saprobes, endophytes and pathogens. A few species have been documented as being etiologic agents in vertebrate hosts (David 1997; Bensch et al. 2012, 2015; Crous et al. 2014). In this study, a new species of *Cladosporium* is described, with high bootstrap support in the phylogenetic analysis (Figure 7).

####### Cladosporium endophyticum

Taxon classificationFungiCapnodialesCladosporiaceae

Tibpromma & K.D. Hyde sp. nov.

MB823838

Figure 8

######## Etymology.

named after its status as an endophytic fungus.

######## Holotype.

MFLU 18-0005

######## Description.

*Colonies* on PDA attaining 9 cm diam. in six weeks at room temperature, slow growing, dark olivaceous. *Mycelium* superficial and immersed composed of septate, branched, 2.3–4.5 µm wide, sub-hyaline, with smooth and thick-walled hyphae. **Sexual morph** Undetermined. **Asexual morph** *Conidiophores* 6–10 µm high, 3–4 µm diam. (x̄ = 8.24 × 3.52 µm, n = 10), terminal and intercalary, cylindrical or sub-cylindrical, darkened conidiogenous loci. *Conidia* 3–6 × 2–4 µm (x̄ = 3.64 × 2.75 µm, n = 30), forming long branched chains, hyaline to pale-olivaceous, smooth and thin-walled, aseptate, globose to ovoid with rounded ends.

######## Culture characteristics.

**Figure 8.**
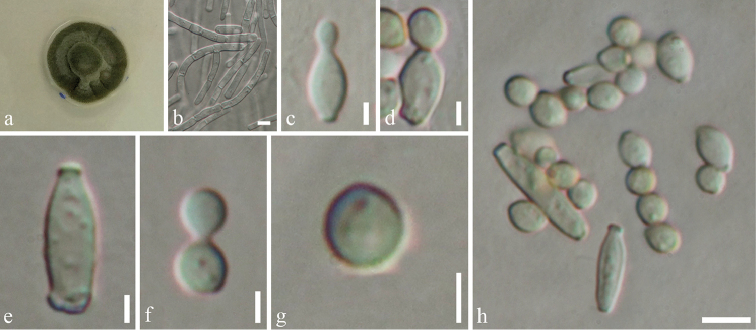
*Cladosporium endophyticum* (MFLU 18-0005, holotype). **a** Colony on MEA media **b** Mycelium masses **c–e** Conidia and conidiogenous cells **f, g** Conidia **h** Conidia and conidiogenous cells. Scale bars: 5 µm (**b–h**), 10 µm (**h**).

Colonies on PDA (Figure 2, PE58), superficial, dark olivaceous with dark-grey centre, irregular, undulate with wrinkled and raised on surface media; reverse dark olivaceous. Generative hyphae simple-septate, branched, sub-hyaline, guttules, thick-walled (Figure 8).

######## Material examined.

THAILAND, Chumphon, Pathio District, on healthy leaves of *Pandanus* sp. (Pandanaceae), 1 December 2016, S. Tibpromma PE58 (MFLU 18-0005, **holotype**); HKAS100855, paratype, ex-type living cultures, MFLUCC 17-0599 = KUMCC 17-0294.

######## GenBank numbers.

ITS=MG646956, LSU=MG646949, SSU=MG646981, TEF1=MG646988.

######## Notes.

*Cladosporium endophyticum* was isolated as an endophyte from *Pandanus* sp. in Thailand. In the phylogenetic analysis of combined gene sequence data of ITS, LSU, SSU and TEF1, the new taxon *Cladosporium endophyticum* is sister to *C. halotolerans* (Figure 7), but well-separated with high bootstrap support (90% in ML). Moreover, the morphology of this new taxon was compared with *Cladosporium halotolerans* which has brown to dark brown, subglobose to globose with verrucose, less often short-ovoid conidia, narrower at both ends (Zalar et al. 2007), while *C. endophyticum* has globose to ovoid, hyaline to pale-olivaceous conidia with rounded ends. Here, the authors introduce the new species *C. endophyticum* and provide an updated phylogenetic tree for the genus *Cladosporium*.

##### Pleosporales Luttr. ex M.E. Barr, 1987

###### Massarinaceae

Taxon classificationFungiPleosporalesMassarinaceae

Munk.

####### Remarks.

The family *Massarinaceae* was introduced by Munk (1956) under *Pleosporales* together with *Cucurbitariaceae* and *Didymosphaeriaceae*. Later, Barr (1987) segregated *Massarinaceae* under *Lophiostomataceae* based on morphology, while based on multigene phylogenetic analysis Schoch et al. (2009) also showed *Massarinaceae* is a distinct family in order *Pleosporales*. Recently, Zhang et al. (2009, 2012) recognised *Massarinaceae* as a distinct lineage based on both morphology and molecular phylogeny. In this study, a new species of endophytic *Massarina*, based on morphological and phylogenetic support, is introduced from *Pandanus* sp. in Thailand.

###### Massarina pandanicola

Taxon classificationFungiPleosporalesMassarinaceae

Tibpromma & K.D. Hyde sp. nov.

MB823839

Figure 10

####### Etymology.

name referring to the host genus of the plant on which the fungus was first discovered (*Pandanus*).

####### Holotype.

MFLU 18-0004

####### Description.

*Colonies* on PDA attaining 9 cm diam. in 4 weeks at room temperature, slow growing, white to yellow-white. *Mycelium* superficial and immersed composed of septate, branched, 2.5–7 µm wide, sub-hyaline, with smooth and thick-walled hyphae. **Sexual morph** Undetermined. **Asexual morph** *Conidiophores* 12–25 µm high, 8–14 µm diam. (x̄ = 15.12 × 10.45 µm, n = 10), enteroblastic, phialidic, cylindrical or sub-cylindrical, sub-hyaline. *Conidia* 3–5 × 1–3 µm (x̄ = 4.34 × 1.75 µm, n = 30), cylindrical, hyaline, smooth and thin-walled, aseptate, rounded ends, guttulate, without sheet or appendages.

####### Culture characteristics.

Colonies on PDA (Figure 2, PE52), superficial, white to yellow-white, irregular, undulate with smooth and raised on surface media; reverse yellow-white. Generative hyphae simple-septate, branched, sub-hyaline, with guttulate cells, thin-walled. Sporulating in culture within 3 months (Figure 10).

####### Material examined.

THAILAND, Chumphon, Pathio District, on healthy leaves of *Pandanus* sp. (Pandanaceae), 1 December 2016, S. Tibpromma PE52 (MFLU 18-0004, **holotype**); HKAS100854, paratype, ex-type living cultures, MFLUCC 17-0596 = KUMCC 17-0293.

####### Genbank numbers.

ITS=MG646958, LSU=MG646947, SSU=MG646979, TEF1=MG646986.

####### Notes.

**Figure 9.**
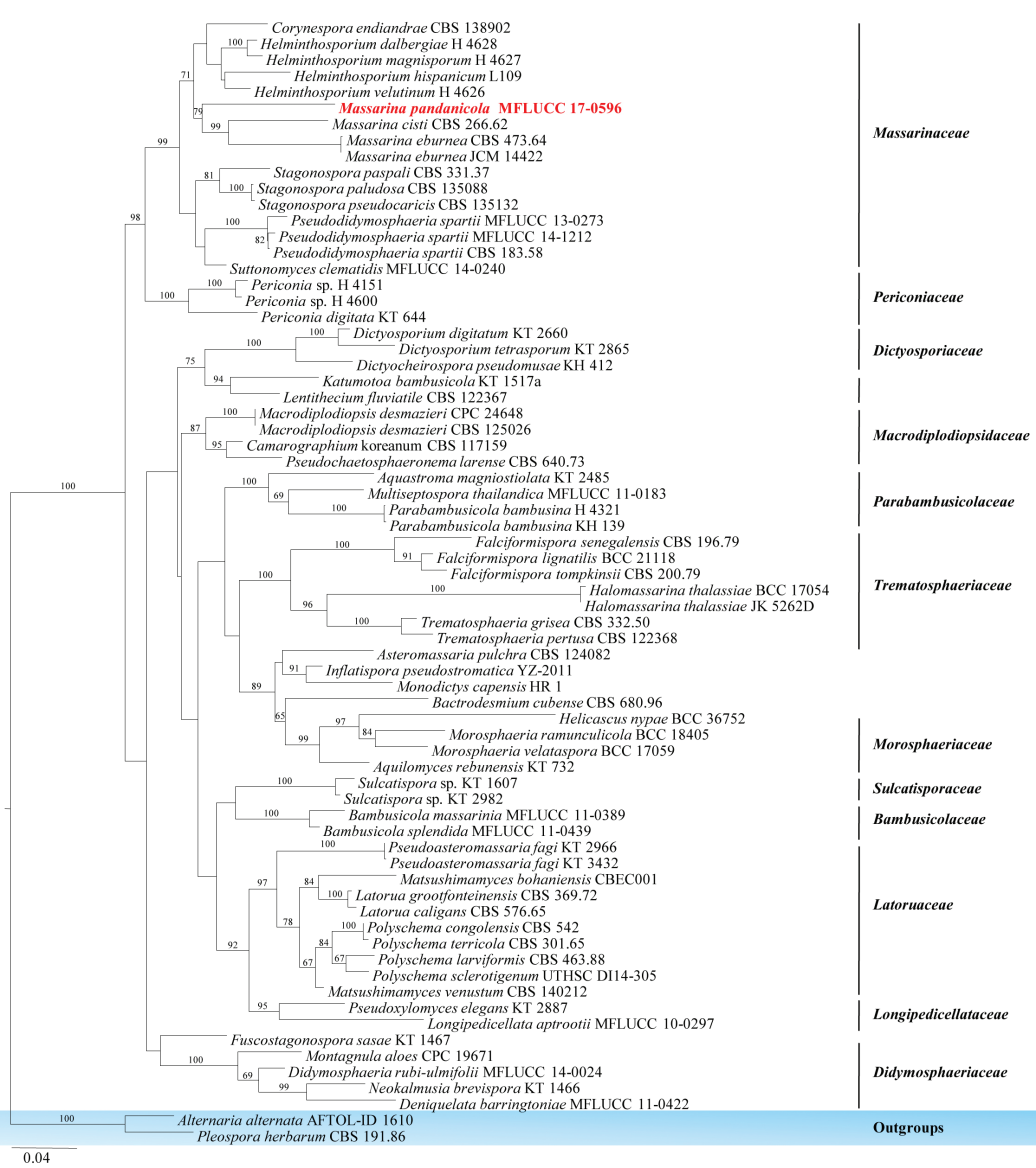
Phylogram generated from maximum likelihood analysis based on ITS, TEF1, SSU, LSU and RPB2 sequenced data. Maximum likelihood bootstrap values are given above/below the nodes. The newly generated sequences in red bold. The tree is rooted with *Alternaria alternata* and *Pleospora herbarum*.

The genus *Massarina* has been known as a phylogenetically diverse group in the order Pleosporales based on molecular data (Liew et al. 2002) and most members in *Massarina* except for the type species (*M. eburnea*) are morphologically variable. The taxon, *Massarina pandanicola* collected from *Pandanus* sp. in Thailand is introduced here as a new species with both morphology and phylogeny support. The morphology of the taxon showed similar conidia with *Massarina eburnean* (Tanaka et al. 2015), but based on phylogenetic analysis of combined ITS, LSU, SSU and TEF1 gene sequence data, the new taxon *M. pandanicola* is well-separated from other species in *Massarina* (Figure 9) with high bootstrap support (79 % in ML). This is the first record of *Massarina* from *Pandanus* sp.

**Figure 10.**
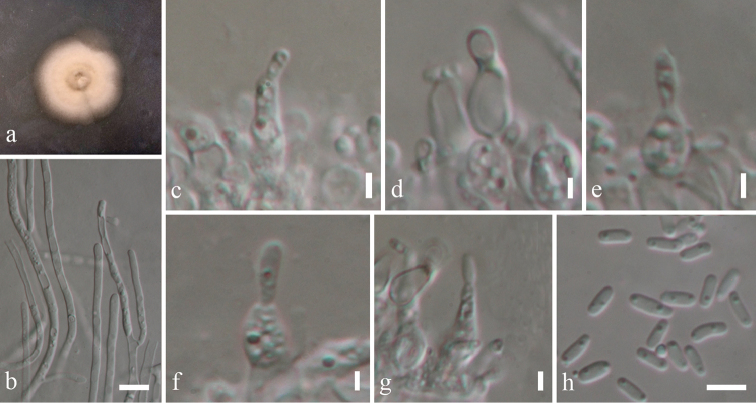
*Massarina pandanicola* (MFLU 18-0004, holotype). **a** Colony on MEA media **b** Mycelium masses **c–g** Conidia and conidiogenous cells **h** Conidia. Scale bars: 20 μm (**b**), 2 μm (**c–g**), 5 μm (**h**).

###### Pleosporaceae

Taxon classificationFungiPleosporalesPleosporaceae

Nitschke

####### Remarks.

The family *Pleosporaceae* was introduced by Nitschke (1869) and is the largest family of the order *Pleosporales* (Hyde et al. 2013; Ariyawansa et al. 2015b; Liu et al. 2017). Members of this family can be endophytes, aquatic or terrestrial saprobes, plant pathogens or opportunistic animal pathogens (Sivanesan 1984; Carter and Boudreaux 2004). A backbone tree for *Pleosporaceae* was provided by Ariyawansa et al. (2015a). In this study, *Alternaria burnsii* is reported from a *Pandanus* sp. host in Thailand.

###### Alternaria burnsii

Taxon classificationFungiPleosporalesPleosporaceae

Uppal, Patel & Kamat, Indian J. Agric. Sci. 8: 49 (1938)

####### Culture characteristics.

Colonies on PDA (Figure 2, PE26), superficial, white-orange to cream, circular, entire edge, smooth, flossy, velvety and raised on surface media; reverse yellow-white at the margin and yellow-brown in centre. Not sporulating in culture.

####### GenBank numbers.

ITS=MG646973, LSU=MG646952, TEF1=MG646987.

####### Notes.

**Figure 11.**
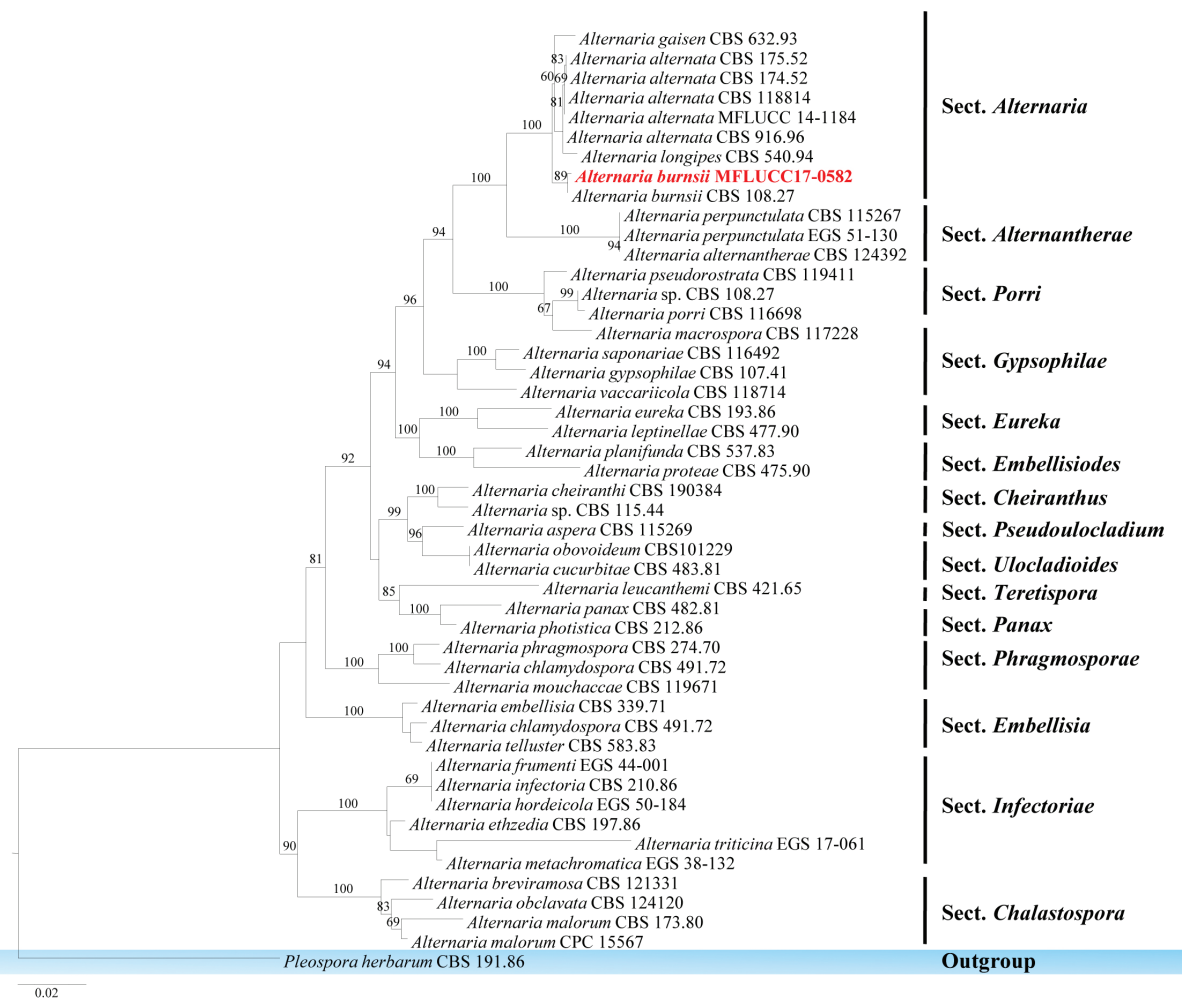
Phylogram generated from maximum likelihood analysis based on ITS, TEF1, LSU and RPB2 sequence data. Maximum likelihood bootstrap values are given above/below the nodes. The newly generated sequences are in red bold. The tree is rooted with *Pleospora herbarum*.

*Alternaria burnsii* was introduced by Uppal et al. (1938) from India on *Cumnium cyminum*. This species has a close phylogenetic relationship with *Alternaria tomato* and *A. jacinthicola* (Woudenberg et al. 2015). Results from phylogenetic analysis show that the authors’ collection belongs to *Alternaria burnsii* with a relatively high bootstrap support (89% in ML) (Figure 11). Nucleotides across the ITS regions of *Alternaria burnsii* CBS 108.27 and the isolates were compared and the authors noted that they are identical.

#### Sordariomycetes O.E. Erikss. & Winka

##### Diaporthales Nannf.

###### Diaporthaceae

Taxon classificationFungiDiaporthalesDiaporthaceae

Höhn. ex Wehm.

####### Remarks.

The family *Diaporthaceae* was introduced by von Höhnel (1917) and was placed in the order Diaporthales. This family comprised two *Diaporthe* genera (*Phomopsis* and *Mazzantia*) (Wehmeyer 1975; Castlebury et al. 2002). Later, *Diaporthaceae* was given the synonym *Valsaceae* (Barr 1978). Based on DNA sequence data, some other genera have been placed in *Diaporthaceae* (Dai et al. 2014; Voglmayr and Jaklitsch 2014). Recently, Maharachchikumbura et al. (2015) and Senanayake et al. (2017) listed further genera that belong to *Diaporthaceae*. In this study, a new and a known species of *Diaporthe* from Pandanaceae hosts in Thailand is reported.

###### Diaporthe pandanicola

Taxon classificationFungiDiaporthalesDiaporthaceae

Tibpromma & K.D. Hyde sp. nov.

MB823840

Figure 13

####### Etymology.

Name referring to the host genus on which the fungus was first discovered (*Pandanus*).

####### Holotype.

MFLU 18-0006

####### Culture characteristics.

Colonies on PDA (Figure 2, PE77), superficial, white, circular with entire edge, smooth and raised on surface media, flossy and velvety; reverse yellow-white, 9 cm diam. in 10 days. Generative hyphae simple-septate, branched, sub-hyaline, cells with guttules, thin-walled, 1.5–7 µm wide. Not sporulating in culture (Figure 13).

**Figure 12.**
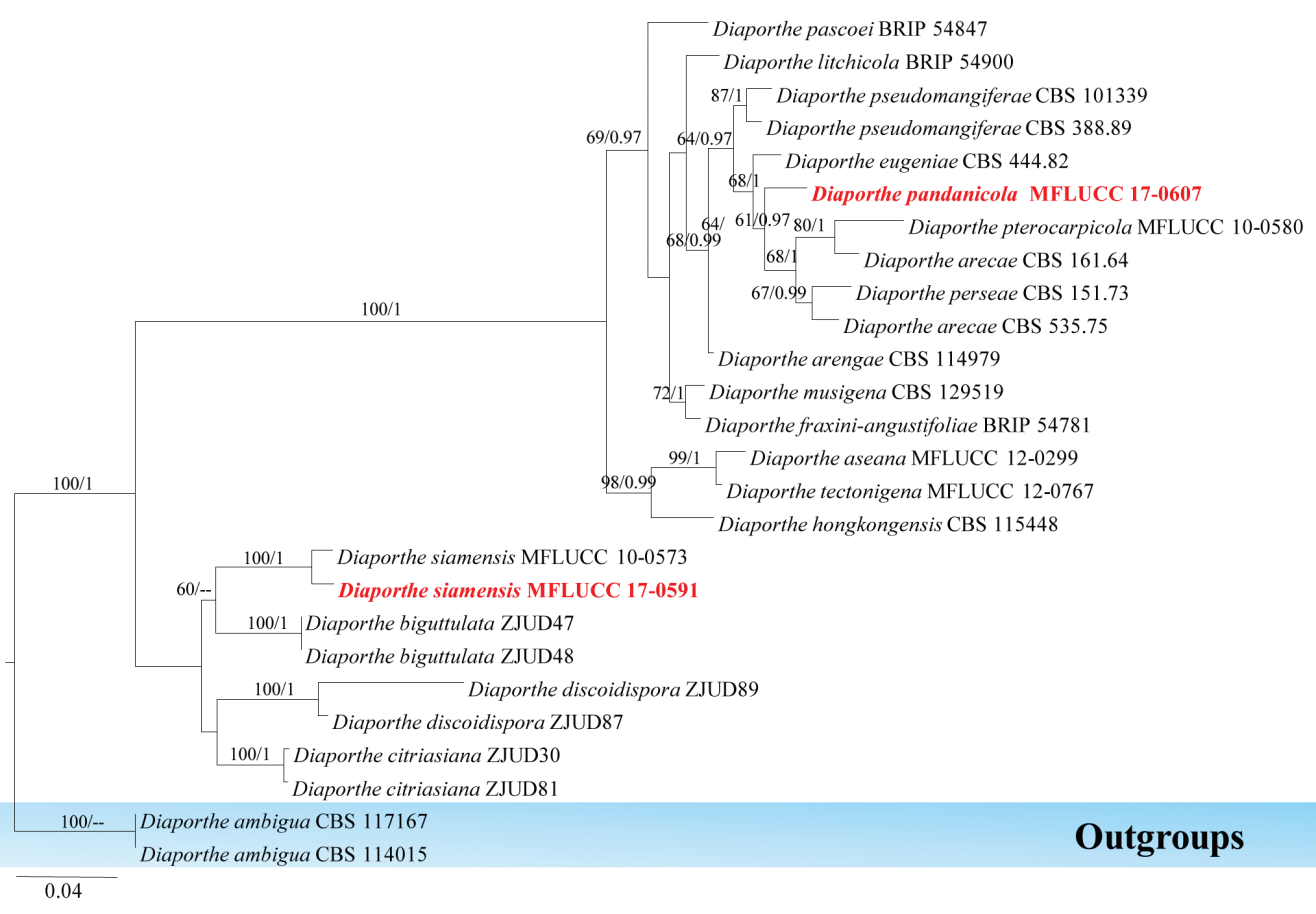
Phylogram generated from maximum likelihood analysis based on ITS, TEF1 and β-tubulin sequenced data. Maximum likelihood (left) and Bayesian inference (right) bootstrap values are given above/below the nodes. The newly generated sequences are in red bold. The tree is rooted with *Diaporthe ambigua*.

**Figure 13.**
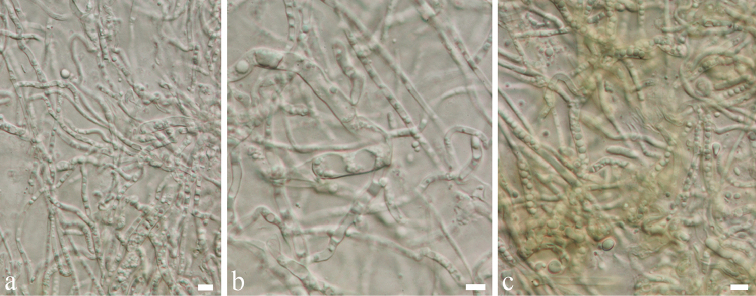
*Diaporthe pandanicola* (MFLU 18-0006, holotype). **a–c** Mycelia masses. Scale bars: 5 µm (**a–c**).

####### Material examined.

THAILAND, Chumphon, Pathio District, on healthy leaves of *Pandanus* sp. (Pandanaceae), 1 December 2016, S. Tibpromma PE77 (MFLU 18-0006, **holotype**); HKAS100858, paratype, ex-type living cultures, MFLUCC 17-0607 = KUMCC 17-0297.

####### GenBank numbers.

ITS=MG646974, β-tubulin=MG646930, ACT=MG646930.

####### Notes.

*Diaporthe* species are plant pathogens, endophytes or saprobes (Carroll 1986; Garcia-Reyne et al. 2011; Udayanga et al. 2011, 2012, 2014, Hyde et al. 2014). Here, a new species *Diaporthe pandanicola* is introduced based on phylogeny support. Based on phylogenetic analysis, the new species was well-separated from closely related species of *Diaporthe* (61% in ML, 0.97 in PP). However, this isolate is an endophytic fungus and did not sporulate in culture during 5 months (Figure 13).

###### Diaporthe siamensis

Taxon classificationFungiDiaporthalesDiaporthaceae

Udayanga, X.Z. Liu & K.D. Hyde, 2012

####### Culture characteristics.

Colonies on PDA (Figure 2, PE37), superficial, white to yellow-white, irregular, curled and raised on media surface, flossy; under surfaceyellow-white.

####### GenBank numbers.

ITS=MG646975, TEF1=MG646989, β-tubulin=MG646925, ACT=MG646940.

####### Notes.

In the phylogenetic analysis, the authors’ collection grouped with *Diaporthe siamensis* MFLUCC 10-0573 with high statistical values of 100% in ML and 1.00 in PP. *Diaporthe siamensis* is an endophytic fungus collected from a Pandanaceae host in Thailand.

##### Glomerellales Chadef. ex Réblová et al.

###### Glomerellaceae

Taxon classificationFungiGlomerellalesGlomerellaceae

Locq. ex Seifert & W. Gams, in Zhang et al. (2007)

####### Remarks.

The family *Glomerellaceae* was introduced by Locquin (1984), but was invalidly published. To date, most *Glomerellaceae* have been recorded to be pathogens (Maharachchikumbura et al. 2016b). Earlier studies reported that the position of the family *Glomerellaceae* was not stable (Zhang et al. 2006; Kirk et al. 2001; Kirk et al. 2008). Réblová et al. (2011) resolved the placement of *Glomerellaceae* by using phylogenetic analysis of combined ITS, LSU, SSU and RPB2 sequence data. Recently, the family *Glomerellaceae* was established based on the genus *Glomerella* (Zhang et al. 2006), which had been given a synonym under its asexual morph *Colletotrichum* (Maharachchikumbura et al. 2015). Recently, Jayawardena et al. (2016) provided notes on currently accepted species of *Colletotrichum*. In this study, the authors introduce a new endophytic *Colletotrichum* species and report a known species of endophytic *Colletotrichum* from gloeosporioides species complex based on morphology and phylogenetic analysis.

###### Colletotrichum fructicola

Taxon classificationFungiSordariomycetesGlomerellaceae

Prihast., L. Cai & K.D. Hyde, 2009

####### Culture characteristics.

Colonies on PDA (Figure 2, PE84, 88), superficial, white to olivaceous in the beginning and later become olivaceous to dark-olivaceous, circular, entire edge, smooth, dense and raised on surface media; reverse dark-olivaceous. Sporulating in culture after 1 month.

####### GenBank numbers.

MFLUCC 17-0613 ITS=MG646968, β-tubulin=MG646927, GAPDH=MG646932, CHS-1=MG646937, ACT=MG646939. MFLUCC 17-0555 ITS=MG646969, β-tubulin=MG646928, GADPH=MG646933, CHS-1=MG646936, ACT=MG646944.

####### Notes.

The gloeosporioides species complex is mainly plant pathogens (Weir et al. 2012) and some species are endophytes (Liu et al. 2015). *Colletotrichum fructicola* has a wide host range (Weir et al. 2012) and was originally reported from coffee berries in Thailand (Prihastuti et al. 2009). In this study, the authors followed Jayawardena et al. (2016) and identify the collection as *Colletotrichum fructicola* which was isolated from a Pandanaceae host. Based on phylogenetic analysis, this taxon grouped with *Colletotrichum fructicola* with 90 % in ML and 1.00 in PP. The ITS, β-tubulin, GAPDH, CHS-1 and ACT DNA nucleotide comparison showed that the taxon and other strains of *Colletotrichum fructicola* Prihast., L. Cai & K.D. Hyde have 100% similarity.

###### Colletotrichum pandanicola

Taxon classificationFungiGlomerellalesGlomerellaceae

Tibpromma & K.D. Hyde sp. nov.

MB823841

Figure 15

####### Etymology.

name referring to the host genus (*Freycinetia*).

####### Holotype.

MFLU 18-0003

####### Description.

Colonies on PDA attaining 9 cm diam. in 7 days at room temperature, dark-grey. **Sexual morph** Undetermined. **Asexual morph** *Conidiophores* hyaline, smooth-walled, cylindrical to slightly inflated. *Conidia* 9–18 µm high, 4–8 µm diam. (= 13.39 × 5.35 µm, n = 20), hyaline, cylindrical with rounded ends tapering slightly towards the base, smooth, septate, guttulate.

####### Culture characteristics.

Colonies on PDA (Figure 2, PE09), superficial, white in the beginning and later becoming dark-grey, circular, entire edge, smooth, flossy, velvety and raised on surface media; reverse dark. Sporulating in culture after 1 month.

####### Material examined.

THAILAND, Chumphon, Pathio District, on healthy leaves of *Pandanus* sp. (Pandanaceae), 1 December 2016, S. Tibpromma PE09 (MFLU 18-0003, **holotype**); GZAAS 16-0145, paratype, ex-type living cultures, MFLUCC 17-0571.

####### GenBank numbers.

ITS=MG646967, β-tubulin=MG646926, GAPDH= MG646931, CHS-1=MG646935, ACT=MG646938.

####### Notes.

**Figure 14.**
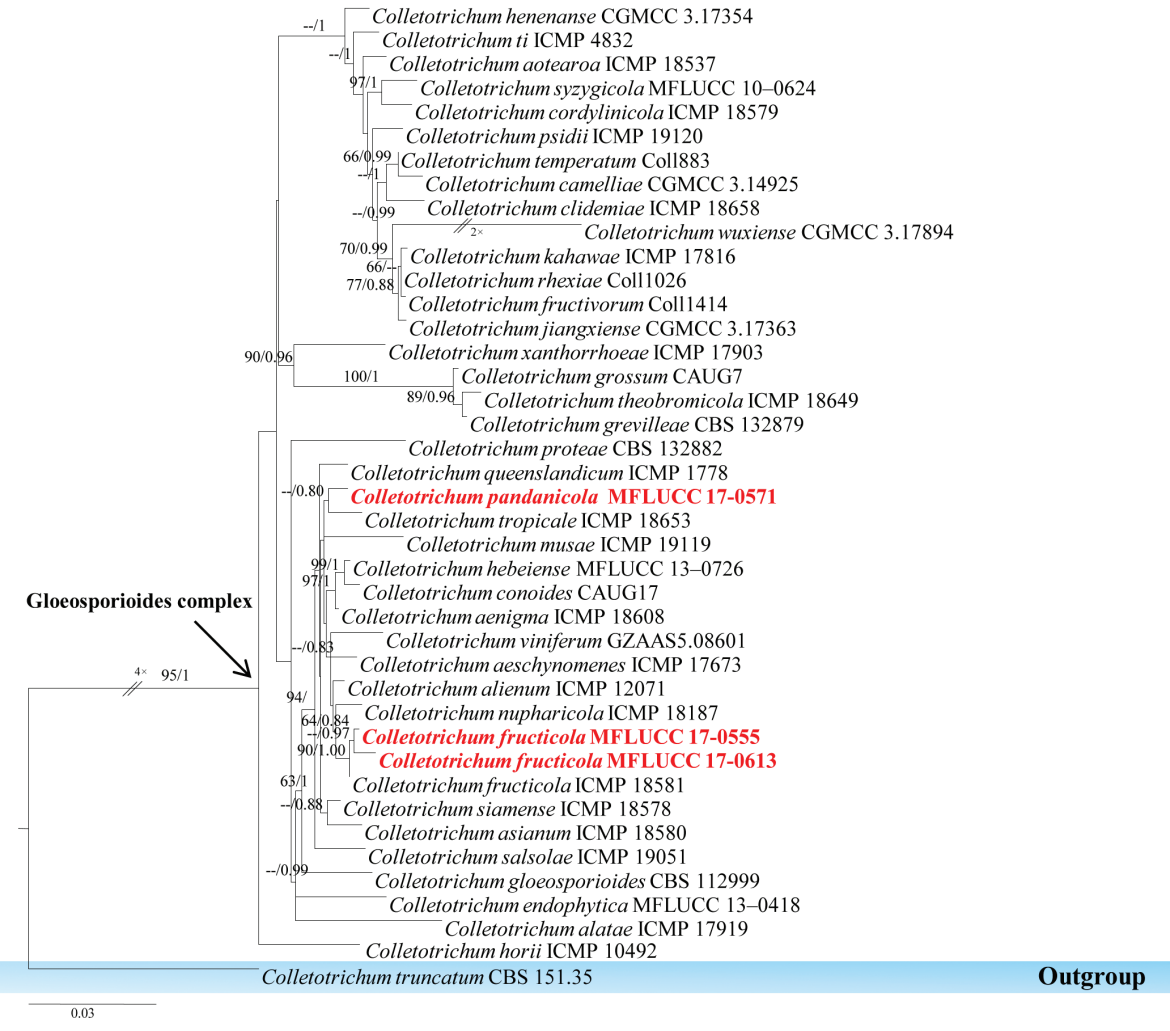
Phylogram generated from maximum likelihood analysis based on combined ITS, Actin, β-tubulin, GADPH and CHS-1 sequenced data. Maximum likelihood (left) and Bayesian inference (right) bootstrap values are given above/below the nodes. The newly generated sequences are in red text. The tree is rooted with *Colletotrichum truncatum*.

*Colletotrichum pandanicola* is introduced here as a new species in the gloeosporioides species complex based on morphological and phylogenetic data. The phylogenetic analysis shows that this new taxon is well-separated from other known *Colletotrichum* species (Figure 14). The authors also compared nucleotides of β-tubulin, GAPDH, CHS-1 and ACT and found that there are differences between *Colletotrichum tropicale* and this new species (β-tubulin 7 bp, GAPDH 11 bp, CHS-1 7 bp and ACT 3 bp).

###### Magnaporthaceae

Taxon classificationFungiMagnaporthalesMagnaporthaceae

P.F. Cannon

####### Remarks.

The family *Magnaporthaceae* was introduced by Cannon (1994) and was placed as a family within the class Sordariomycetes (Kirk et al. 2001; Lumbsch and Huhndorf 2007). According to Thongkantha et al. (2009), the placement of the taxa *Magnaporthaceae* has long been problematic due to a lack of convincing morphological characteristics and inconclusive molecular data. Thongkantha et al. (2009) established a new order, *Magnaporthales*, to accommodate *Magnaporthaceae*, based on a combination of morphological characteristics and the phylogenetic analysis of combined sequence data. Maharachchikumbura et al. (2015) provided an updated outline of the family *Magnaporthacea*e with 20 genera, which included both sexual and asexual morphs. In this study, *Mycoleptodiscus endophyticus* is introduced as a new species.

###### Mycoleptodiscus endophyticus

Taxon classificationFungiMagnaporthalesMagnaporthaceae

Tibpromma & K.D. Hyde sp. nov.

MB823842

Figure 17

####### Etymology.

Named after its original habitat as an endophytic fungus.

####### Holotype.

MFLU 18-0001

####### Culture characteristics.

Colonies on PDA (Figure 2, FE101), superficial, dark olivaceous with circular rings with filiform edge and rough and raised on media surface; reverse dark olivaceous. *Mycelium* composed of branched, pale-brown to dark-brown, thick-walled, guttulate, hyphae, with cells sub-globose to ovoid in shape. Not sporulating in culture.

####### Material examined.

THAILAND, Ranong, Muang, on healthy leaves of *Freycinetia* sp. (Pandanaceae), 3 December 2016, S. Tibpromma FE101 (MFLU 18-0001, **holotype**); HKAS100847, paratype, ex-type living cultures, MFLUCC 17-0545 = KUMCC 17-0263.

####### GenBank numbers.

LSU=MG646946, SSU=MG646978, TEF1=MG646985.

####### Notes.

*Mycoleptodiscus* Ostaz. (1968) belongs to *Magnaporthaceae*, *Magnaporthales.* Since 1968, there have been 17 records of *Mycoleptodiscus* in Index Fungorum. Most of these species were described without molecular data. In this study, a new species *Mycoleptodiscus endophyticus* is introduced, based on culture characteristics and phylogenetic analysis (100 % in ML). *Mycoleptodiscus endophyticus* was found as an endophytic fungus on leaves of *Freycinetia* sp; *Mycoleptodiscus freycinetiae* Whitton, K.D. Hyde & McKenzie was found as a saprobic fungus on the same host but there was no molecular data available to confirm this identification. The authors were unable to compare the morphological differences between the new taxon and *Mycoleptodiscus freycinetiae*, because only culture characteristics are presented here for this new taxon (Fig. 17).

**Figure 15.**
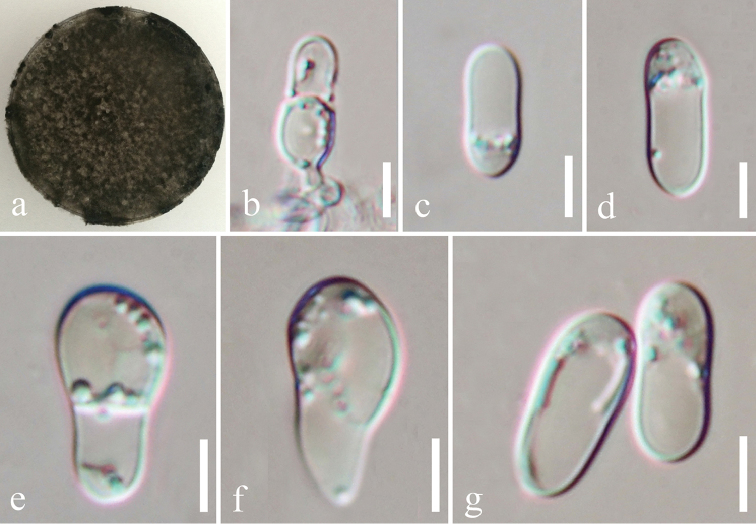
*Colletotrichum pandanicola* (MFLU 18-0003, holotype). **a** Colony on PDA media **b** Conidia and conidiogenous cells **c–g** Conidia on PDA culture. Scale bars: 5 μm (**b**), 2 μm (**c–g**).

**Figure 16.**
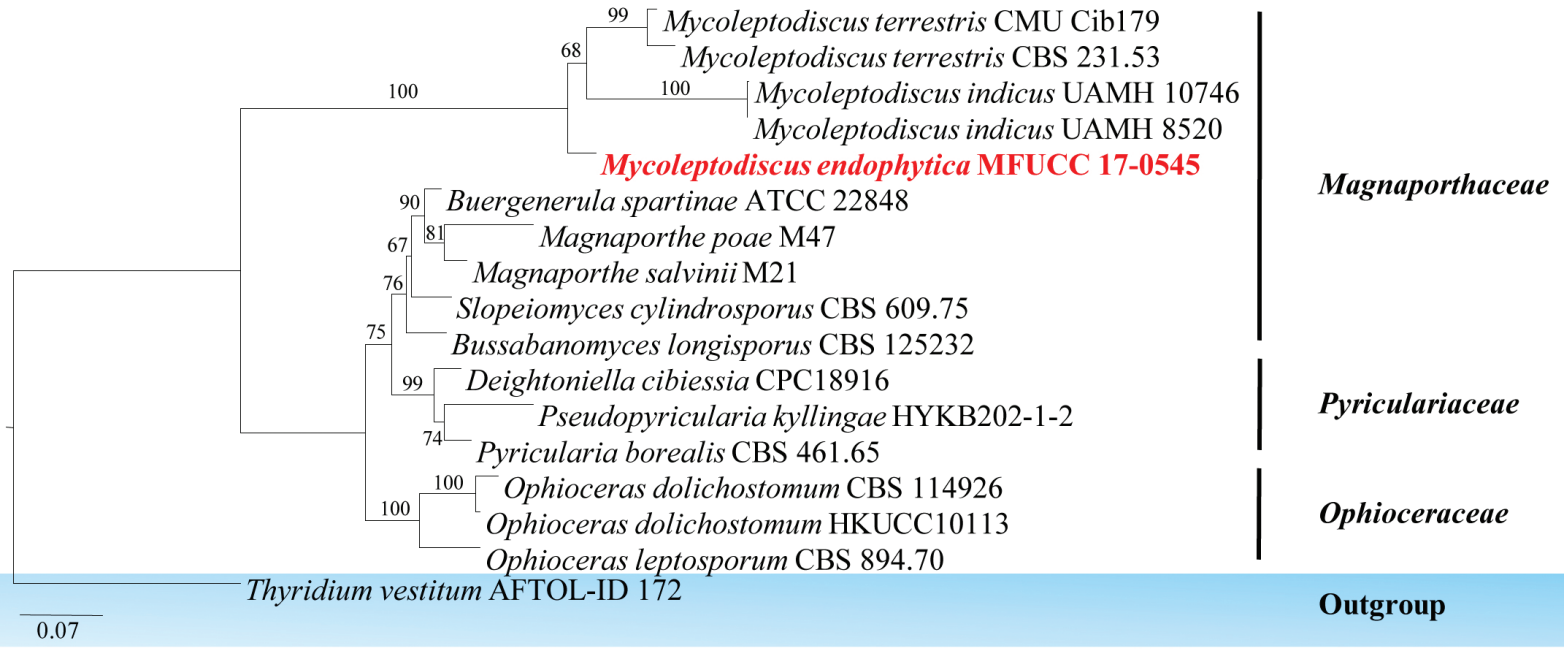
Phylogram generated from maximum likelihood analysis based on combined ITS, LSU, SSU and TEF1 sequenced data. Maximum parsimony bootstrap values are given above/below the nodes. The newly generated sequences are in red bold. The tree is rooted with *Thyridium vestitum*.

**Figure 17.**
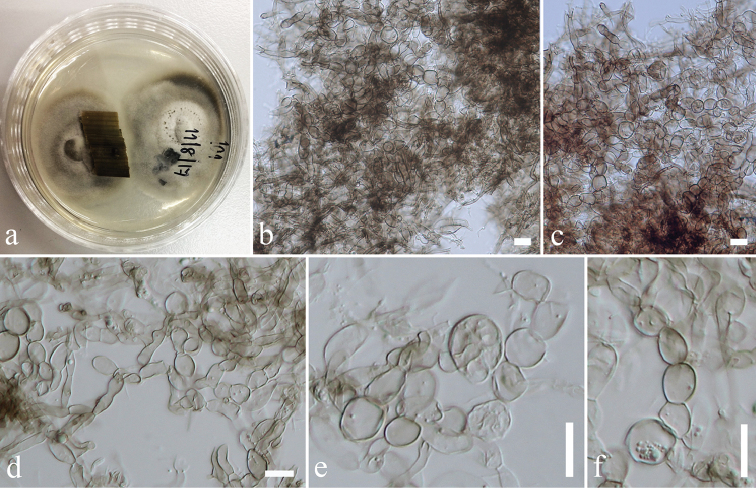
*Mycoleptodiscus endophyticus* (MFLU 18-0001, holotype). **a** Colony on MEA media **b, c** Mycelia masses **d–f** Vegetative hyphae in culture. Scale bars: 10 μm (**b–d**), 5 μm (**e, f**).

###### Sporocadaceae

Taxon classificationFungiXylarialesSporocadaceae

Corda, 1842

####### Remarks.

*Sporocadaceae* was introduced by Corda (1842) with *Pestalotiopsis*-like asexual morphs and confirmed by Senanayake et al. (2015). Members of *Sporocadaceae* are saprobes, endophytes or foliar pathogens in tropical and temperate regions (Jeewon et al. 2004; Tanaka et al. 2011). *Pestalotiopsis* can be found as saprobes or pathogens worldwide (Jeewon et al. 2002, 2003; Maharachchikumbura et al. 2011, 2012, 2013, 2014a, b, 2016a, c). Recently, Chen et al. (2017) provided updates for this genus based on morphology and phylogeny. In this study, two known species of *Pestalotiopsis* from Pandanaceae hosts were isolated.

**Figure 18.**
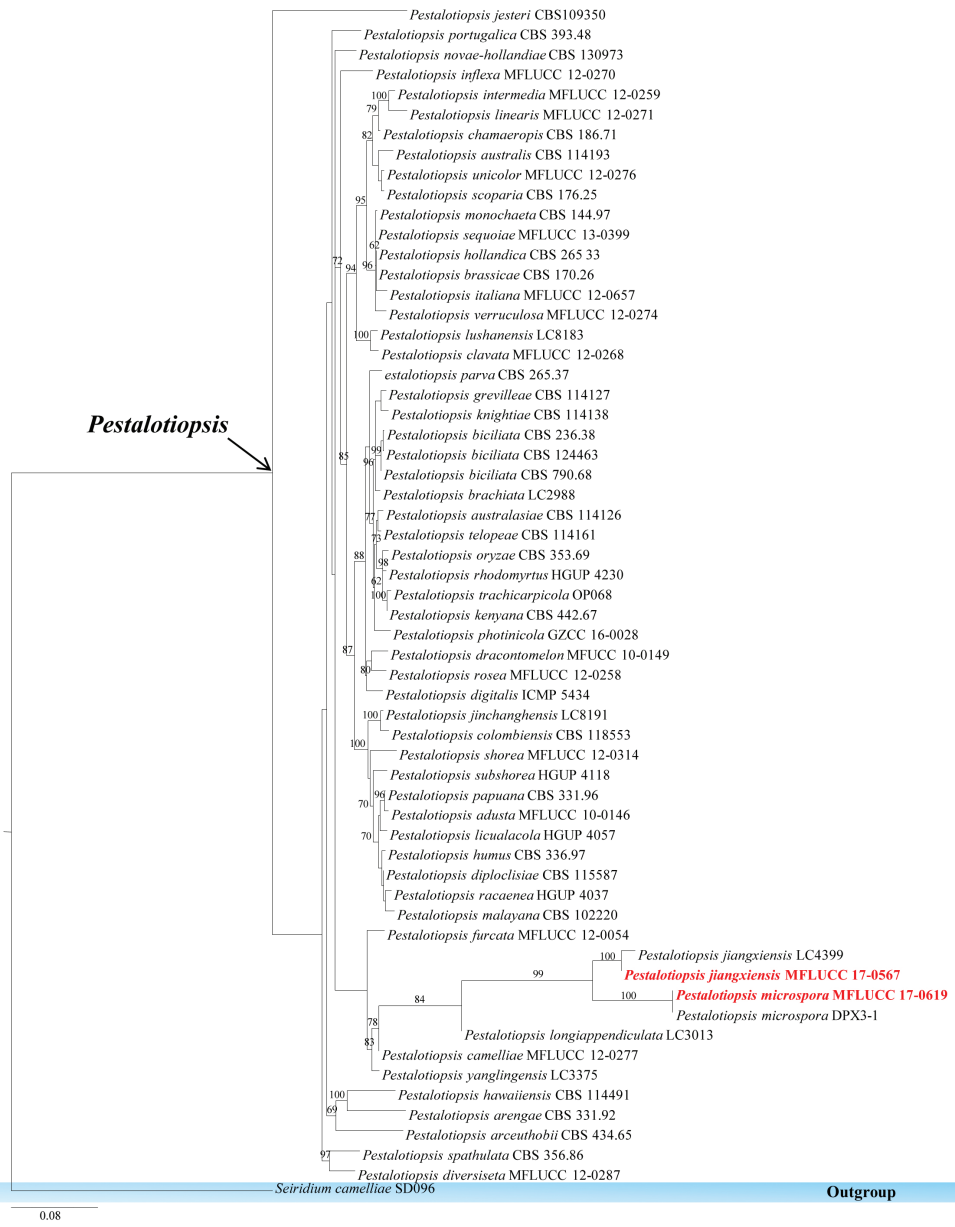
Phylogram generated from maximum likelihood analysis based on the combination of ITS, β-tubulin and TEF1 sequenced data. Maximum parsimony bootstrap is given above/below the nodes. The newly generated sequences are in red bold. The tree is rooted with *Seiridium camelliae*.

###### Pestalotiopsis jiangxiensis

Taxon classificationFungiXylarialesAmphisphaeriaceae

F. Liu & L. Cai, 2017

####### Culture characteristics.

Colonies on PDA (Figure 2, PE05), superficial, white at the margin with yellow-white in the centre, with circular to undulate at the edge and raised and dense aerial mycelia on surface; reverse yellow-white. Sporulating in culture after 2 months.

####### GenBank numbers.

ITS=MG646966, ACT=MG646942, GAPDH=MG646934, β-tubulin=MG646929.

####### Notes.

The authors’ collection from Pandanaceae host in Thailand was identified as *Pestalotiopsis jiangxiensis*. This taxon grouped with *Pestalotiopsis jiangxiensis* LC4399 which is collected from *Eurya* sp., with high bootstrap support of 100% in ML.

###### Pestalotiopsis microspora

Taxon classificationFungiXylarialesAmphisphaeriaceae

(Speg.) G.C. Zhao & N. Li, 1995

####### Culture characteristics.

Colonies on PDA (Figure 2, PE92), superficial, white to yellow-white, edge irregular, flossy and velvety; under surface yellow-white to yellow. Sporulating in culture after 2 months.

####### GenBank numbers.

ITS=MG646965, ACT=MG646943.

####### Notes.

*Pestalotiopsis microspora* was isolated from a Pandanaceae host in Thailand. This strain clusters with *Pestalotiopsis microspora* DPX3-1 with a strong bootstrap support.

#### Saccharomycetes

##### Debaryomycetaceae

Taxon classificationFungiSaccharomycetalesDebaryomycetaceae

Kurtzman & M. Suzuki

###### Remarks.

*Debaryomycetaceae* was introduced by Kurtzman and Suzuki in 2010 and was typified by *Debaryomyces* Klöcker. *Meyerozyma* belongs to family *Debaryomycetaceae* and was detailed in Kurtzman and Suzuki (2010). In this study, *Meyerozyma caribbica* was found on a Pandanaceae host as an endophytic fungus. Species identification was confirmed by DNA sequence data.

**Figure 19.**
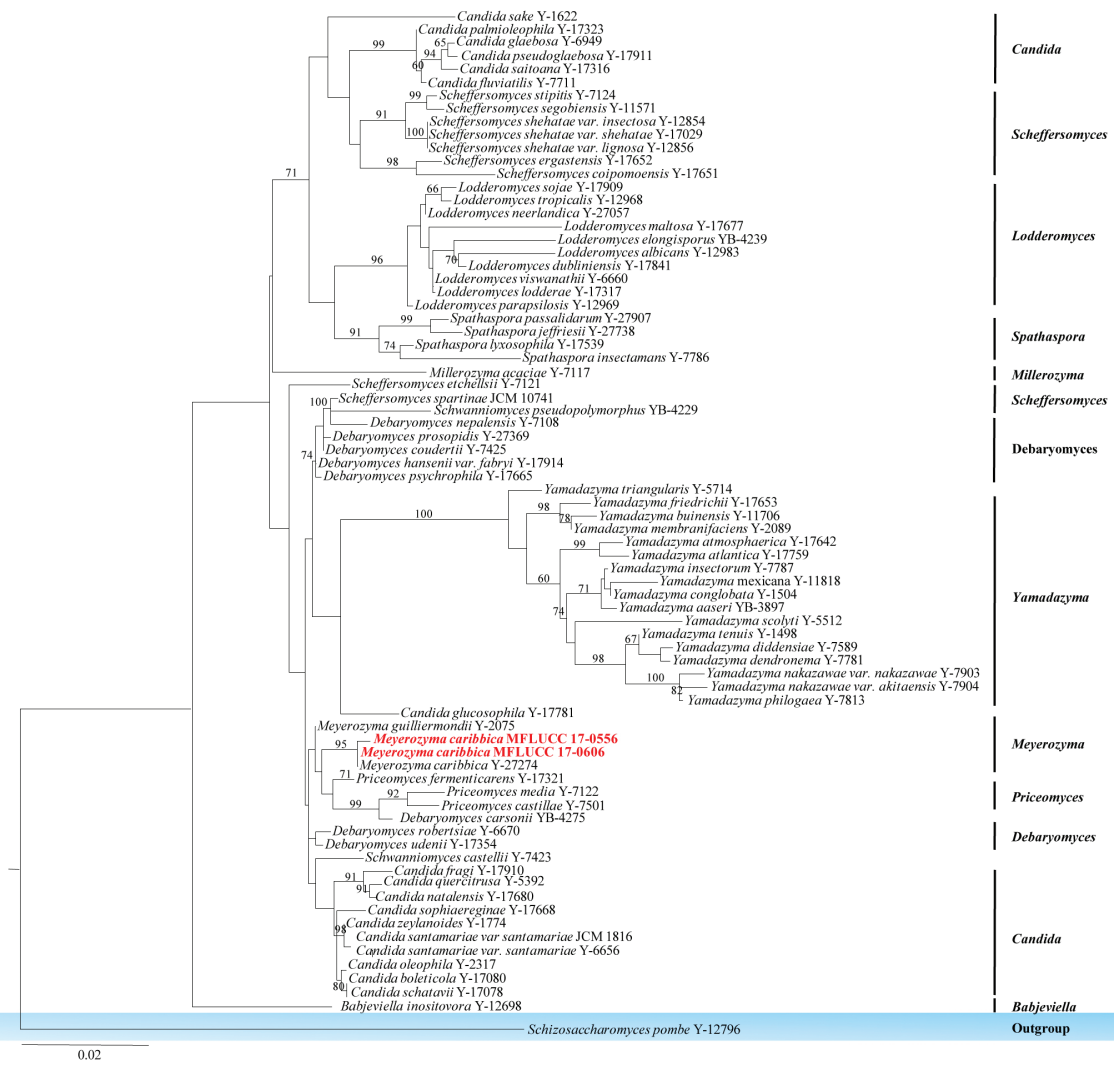
Phylogram generated from maximum likelihood analysis based on combined LSU and SSU sequence data. Maximum parsimony bootstrap is given above/below the nodes. The newly generated sequences are in red text. The tree is rooted with *Schizosaccharomyces pombe*.

##### Meyerozyma caribbica

Taxon classificationFungiSaccharomycetalesDebaryomycetaceae

(Vaughan-Mart., Kurtzman, S.A. Mey. & E.B. O’Neill) Kurtzman & M. Suzuki, Mycoscience 51(1): 8 (2010)

###### Culture characteristics.

Colonies on PDA (Figure 2, PE75, 98), superficial, white to yellow-white, rings with irregular, undulate edge and curled, raised on the surface media; reverse yellow-white to yellow at the margin and dark-brown at the centre. Sporulating in culture after 2 months.

###### GenBank numbers.

MFLUCC 17-0556 ITS=MG646971, LSU=MG646950, SSU=MG646977. MFLUCC 17-0606 ITS=MG646972, LSU=MG646951, SSU=MG646980.

###### Notes.

*Meyerozyma caribbica* collected in this study is represented by two endophytic isolates from Pandanaceae. Phylogenetic analysis also supported the identification of this sample as *Meyerozyma caribbica*.

## Conclusion

In this study on fungal endophytes found on leaves of Pandanaceae, it was found that the taxa belonged to both Ascomycota and Basidiomycota. The majority of the taxa were Ascomycota, as found in most previous endophytic studies (Crozier et al. 2006; Selim et al. 2017). In classical mycology, most endophytic fungi were described based on their morphological features (Barseghyan and Wasser 2010). However, there are difficulties in identifying ascomycetes to the species level based only on morphological features (Lu et al. 2012), because they have only a small set of morphological characteristics and exhibit homoplasy (Barseghyan and Wasser 2010).

The 22 endophytic fungal strains found in this study were chiefly identified using their microscopic characteristics and DNA sequence data and holotype materials in the form of dried cultures. Future studies are however needed to recollect the taxa which are sporulating to describe sexual and asexual characteristics (*sensu* Lacap et al. 2003). In this study, 22 endophytes were isolated and sorted into eight morphotype based on colony characteristics. The authors, however, subjected all isolates to phylogenetic analysis and found they belong to 16 different taxa. The taxa were sorted roughly into morphotypes, but they did not reflect the actual species. Several isolates of this study did not sporulate, but are introduced as new species because DNA sequence comparison and multi-gene phylogenetic analyses provided sufficient evidence to show that they are distinct taxa (Jeewon and Hyde 2016). The new taxa are, however, code compliant, as they are provided with MycoBank numbers, full descriptions, colour photographss and illustrations.

The species composition of endophytic microorganisms is likely to depend on the plant age, genotype, sampled tissue, host type and season of isolation (Rosenblueth and Martinez-Romero 2006). Promputtha et al. (2007) showed that endophytic species can change their ecological strategies and adopt a saprotrophic lifestyle. However, it was found that for the cultures of some endophytic fungal species, mycelia are the only visible morphological structures. According to these conclusions, the authors agree with Petrini (1991), Yanna and Hyde (2002), Ghimire and Hyde (2004) and Hyde et al. (2006) regarding the relationships between fungal endophytes and saprobic fungi. However, the use of next-generation sequencing (NGS) (Shendure and Ji 2008) is another option for identification of fungal species that cannot be cultured *in vitro* and has now become popular. These methods have also been applied to large-scale culture-independent molecular biological methods (Zoll et al. 2016). Future developments in technology are likely to produce further novel methods that mycologists could apply to the field of taxonomy (e.g. Hawksworth and Lucking 2017).

## Supplementary Material

XML Treatment for Polyporaceae

XML Treatment for Endopandanicola

XML Treatment for Endopandanicola thailandica

XML Treatment for Phanerochaete chrysosporium

XML Treatment for Botryosphaeriales

XML Treatment for Endomelanconiopsis freycinetiae

XML Treatment for Phyllosticta capitalensis

XML Treatment for Lasiodiplodia theobromae

XML Treatment for Cladosporium

XML Treatment for Cladosporium endophyticum

XML Treatment for Massarinaceae

XML Treatment for Massarina pandanicola

XML Treatment for Pleosporaceae

XML Treatment for Alternaria burnsii

XML Treatment for Diaporthaceae

XML Treatment for Diaporthe pandanicola

XML Treatment for Diaporthe siamensis

XML Treatment for Glomerellaceae

XML Treatment for Colletotrichum fructicola

XML Treatment for Colletotrichum pandanicola

XML Treatment for Magnaporthaceae

XML Treatment for Mycoleptodiscus endophyticus

XML Treatment for Sporocadaceae

XML Treatment for Pestalotiopsis jiangxiensis

XML Treatment for Pestalotiopsis microspora

XML Treatment for Debaryomycetaceae

XML Treatment for Meyerozyma caribbica
